# C-di-AMP levels modulate *Staphylococcus aureus* cell wall thickness, response to oxidative stress, and antibiotic resistance and tolerance

**DOI:** 10.1128/spectrum.02788-23

**Published:** 2023-11-10

**Authors:** Vanina Dengler Haunreiter, Andrea Tarnutzer, Julian Bär, Manuela von Matt, Sanne Hertegonne, Federica Andreoni, Clément Vulin, Lisa Künzi, Carmen Menzi, Patrick Kiefer, Philipp Christen, Julia A. Vorholt, Annelies S. Zinkernagel

**Affiliations:** 1 Department of Infectious Diseases and Hospital Epidemiology, University Hospital Zurich, University of Zurich, Zurich, Switzerland; 2 Department of Biology, Institute of Microbiology, ETH Zurich, Zurich, Switzerland; University of North Carolina at Chapel Hill, Chapel Hill, North Carolina, USA

**Keywords:** c-di-AMP, *Staphylococcus aureus*, antibiotic tolerance, cell wall thickness, virulence determinants, antibiotic resistance

## Abstract

Beta-lactam antibiotics are widely used to treat infections caused by the important human pathogen *Staphylococcus aureus*. Resistance to beta-lactams, as found in methicillin-resistant *S. aureus*, renders effective treatment difficult. The second messenger cyclic di-3′,5′-adenosine monophosphate (c-di-AMP) promotes beta-lactam resistance in clinical *S. aureus* isolates. C-di-AMP plays a crucial role in the regulation of cellular processes such as virulence, cell wall homeostasis, and resistance to beta-lactams in many bacterial species. In *S. aureus,* c-di-AMP synthesis is mediated by the diadenylate cyclase DacA, while its degradation is carried out by the phosphodiesterases GdpP and Pde2. In this work, we assessed the effect of altered c-di-AMP levels due to mutations in *dacA*, *gdpP,* or *gdpP/pde2* on virulence determinants. We report that a previously described growth defect in bacteria producing high c-di-AMP levels is mainly attributable to smaller cell size. High c-di-AMP levels also led to decreased survival upon oxidative stress, reduced production of the antioxidant staphyloxanthin, increased oxacillin and fosfomycin resistance, and increased cell wall thickness. While resistance to ceftaroline was not affected, high c-di-AMP levels promoted tolerance to this antibiotic. In response to cell wall stress induced by antibiotics, the three-component regulatory system VraTSR mediates an increase in cell wall synthesis via the cell wall stress stimulon (CWSS). Increased c-di-AMP levels led to an activation of the CWSS. Upon deletion of *vraR*, resistance to oxacillin and fosfomycin as well as cell wall thickness diminished in the Δ*gdpP* mutant, indicating a contribution of the VraTSR system to the cell wall related phenotypes.

**IMPORTANCE:**

Antibiotic resistance and tolerance are substantial healthcare-related problems, hampering effective treatment of bacterial infections. Mutations in the phosphodiesterase GdpP, which degrades cyclic di-3′, 5′-adenosine monophosphate (c-di-AMP), have recently been associated with resistance to beta-lactam antibiotics in clinical *Staphylococcus aureus* isolates. In this study, we show that high c-di-AMP levels decreased the cell size and increased the cell wall thickness in *S. aureus* mutant strains. As a consequence, an increase in resistance to cell wall targeting antibiotics, such as oxacillin and fosfomycin as well as in tolerance to ceftaroline, a cephalosporine used to treat methicillin-resistant *S. aureus* infections, was observed. These findings underline the importance of investigating the role of c-di-AMP in the development of tolerance and resistance to antibiotics in order to optimize treatment in the clinical setting.

## INTRODUCTION

Antibiotic resistance and tolerance greatly hinder the effectiveness of bacterial infection treatment. Beta-lactam antibiotics are widely used to treat infections caused by the important human pathogen *Staphylococcus aureus*. However, resistance development as seen in methicillin-resistant *S. aureus* (MRSA) is commonly encountered both in community- and hospital-acquired infections (1).

Second messenger signaling allows bacteria to rapidly respond to environmental changes including antibiotics. Cyclic di-3′,5′-adenosine monophosphate (c-di-AMP) is one of the most recently discovered dinucleotide second messenger and is present in many bacterial species, predominantly in the Gram-positive phyla Bacillota (formerly Firmicutes) and Actinobacteria, but also in some Gram-negative genera and in certain Archaea (2 – 4). In *S. aureus*, c-di-AMP is synthesized by the diadenylate cyclase DacA and degraded by two phosphodiesterases containing a DHH/DHHA1 domain, GdpP (membrane-bound) and Pde2 (cytosolic) (5, 6). GdpP degrades c-di-AMP to pApA in a single-step reaction, while Pde2 converts c-di-AMP and pApA to AMP. Although Pde2 is able to degrade c-di-AMP, Bowman and colleagues found that staphylococcal Pde2 preferably degrades pApA to AMP, while GdpP is mainly responsible for c-di-AMP degradation (6).

C-di-AMP is the only second messenger molecule known to be essential for growth under standard laboratory conditions (7). It is involved in various cellular processes, including virulence, salt and cell wall homeostasis, and resistance to beta-lactam antibiotics (8, 9). In *S. aureus*, c-di-AMP regulates protein activity by binding directly to the protein, e.g., to proteins involved in the potassium regulation (10), while in other species binding to a transcription factor or riboswitch has been shown [reviewed in references (4, 7, 11)]. Various mechanisms can lead to beta-lactam resistance, the most common ones in methicillin-resistant *S. aureus* (MRSA) being the expression of an alternative penicillin-binding protein with reduced affinity to beta-lactams (PBP2a, encoded on *mec*) (1) or increased peptidoglycan crosslinking via upregulation of PBP4 (12, 13). Mutations in *gdpP* were only recently found to confer resistance to beta-lactam antibiotics in clinical isolates lacking *mec* genes and displaying normal PBP4 expression levels, emphasizing the clinical relevance of c-di-AMP levels in conferring resistance to antibiotics (14 – 16).

C-di-AMP is known to influence bacterial cell wall homeostasis; however, the underlying mechanisms are not fully understood and are likely species-specific. In *S. aureus*, high c-di-AMP levels result in increased peptidoglycan crosslinking and resistance to beta-lactam antibiotics, while low c-di-AMP levels decrease beta-lactam antibiotics resistance (6, 17). Cell wall targeting antibiotics can activate the three-component regulatory system VraTSR, which regulates the expression of more than 40 genes belonging to the cell wall stress stimulon (CWSS), leading to increased cell wall synthesis and reduced expression of autolysins (18 – 20). A link between c-di-AMP levels and CWSS activation was found in two different *S. aureus dacA_G206S_* mutants, where decreased c-di-AMP levels correlated with a decreased activation of the CWSS (17).

In this work, we investigated the effect of altered c-di-AMP levels on various *S. aureus* virulence determinants such as growth, oxidative stress response, cell wall thickness, and antibiotic susceptibility as well as the connection with the CWSS.

## RESULTS

### C-di-AMP indirectly influences growth characteristics through cell size

**Fig 1:**
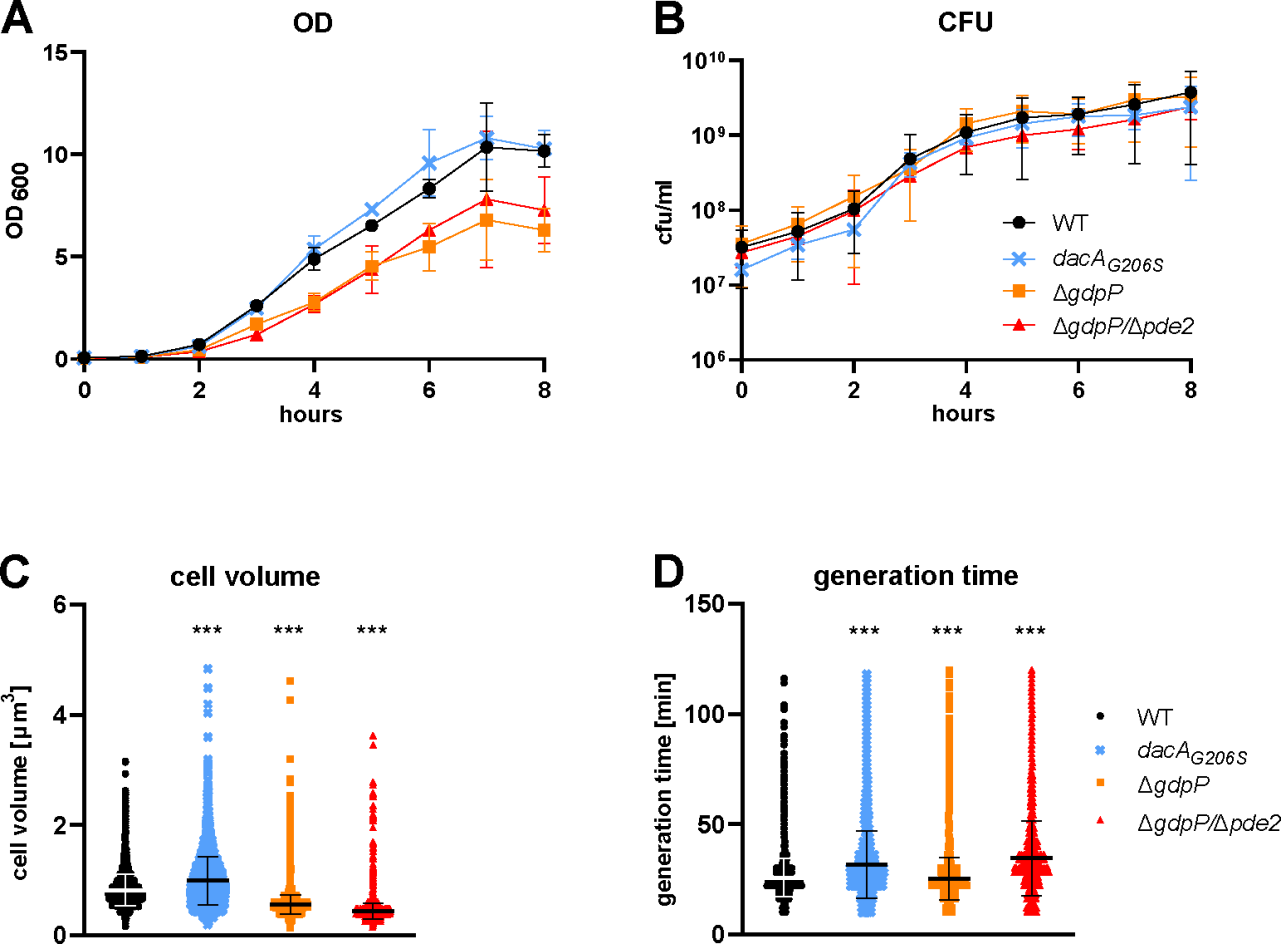
C-di-AMP indirectly influences growth characteristics through cell size. OD (**A**) and CFUs (**B**) were measured in TSB. Data display mean ± SD of three biological replicates. Nonlinear regression showed no significant differences in CFUs. Cell volume (**C**) and generation time (time between division events) (**D**) of single cells were determined in a microfluidic microscope system. Each dot represents one cell, and bars indicate mean ± SD (**C and D**). Statistical significance was assessed by a mixed effects model using a random intercept term for the biological replicate (three replicates) followed by estimated marginal means post-hoc tests comparing WT to each other strain including multivariate *t* distribution based *P*-value correction. ***P < 0.001.

Previous studies have demonstrated in *S. aureus* that the absence of either one or both phosphodiesterases (Δ*gdpP*, *Δpde2*, or Δ*gdpP/*Δ*pde2*) resulted in increased c-di-AMP levels and lowered the growth rate based on optical density (OD) measurements (6, 21). In contrast, a *dacA_G206S_* mutant strain, producing lower amounts of c-di-AMP, grew comparably to the wild-type (WT) strain or slightly faster depending on the *S. aureus* strain background (6, 17, 22). Using the MRSA strain LAC* and its isogenic *dacA_G206S_*, Δ*gdpP*, and Δ*gdpP/*Δ*pde2* mutants, we aimed to further dissect the impact of c-di-AMP levels on various growth characteristics. As expected, c-di-AMP levels were increased in the Δ*gdpP* mutant compared to the WT (Table S1). The Δ*gdpP*/Δ*pde2* mutant showed even higher levels than the Δ*gdpP* single mutant, while c-di-AMP levels decreased in the *dacA_G206S_* strain (Table S1). Measuring OD as a population size proxy, we observed an attenuated increase in OD in the mutants with higher c-di-AMP levels, as described before (6, 21) (Fig. 1A). However, when measuring the colony-forming units (CFUs) after growth in liquid tryptic soy broth (TSB), we detected a similar growth rate in all strains (Fig. 1B). An effect due to viability can be excluded as we found no significant differences in cell viability measured by a live-dead stain with flow cytometry between WT, ∆*gdpP* and ∆*gdpP/*∆*pde2* mutant strains (Fig. S1A).

It has been shown that c-di-AMP plays a role in controlling cell size (5, 22). As OD measurements depend on the cell size (23, 24), we assessed the volume of single cells growing in a mother-machine microfluidic device by single-cell microscopy (representative movie of each strain in Supplementary Material). Compared to the WT (0.82 ± 0.29 µm^3^), the mean cell volume significantly increased in the *dacA_G206S_* mutant (0.99 ± 0.43 µm^3^, 121% of WT) and decreased in the Δ*gdpP* (0.55 ± 0.17 µm^3^, 68% of WT) as well as in the ∆*gdpP/*∆*pde2* (0.43 ± 0.15 µm^3^, 53% of WT) mutant, suggesting that c-di-AMP affects cell volume in a concentration-dependent manner (Fig. 1C). In line with these results, the colony size measured 24 h after inoculation on agar plates negatively correlated with c-di-AMP levels (Fig. S1B). Next, we assessed the generation time (defined as time between cell division events) and found a significantly prolonged generation time in both the *dacA_G206S_* (31.51 ± 15.27 min) and the ∆*gdpP/*∆*pde2* (34.54 ± 16.92 min) mutants compared to the WT (25.68 ± 8.75 min; Fig. 1D). In contrast, the Δ*gdpP* mutant (25.03 ± 9.62 min) showed no indication of impaired growth but a biologically negligible, statistically significant decrease in generation time (0.6 min faster than the WT).

Based on these observations, we conclude that the reduction in OD-based growth in high c-di-AMP mutants is biased by the smaller cell size and does not reflect growth impairment in the sense of a reduction in cell division rate or viability.

### Elevated c-di-AMP levels lead to decreased survival under oxidative stress and reduced staphyloxanthin production

**Fig 2:**
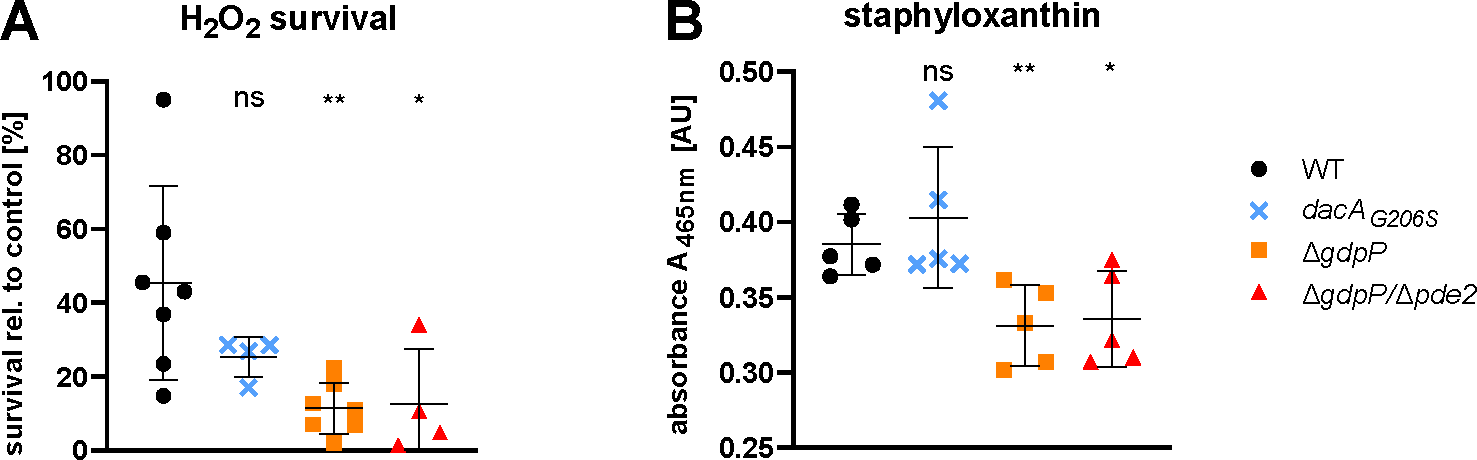
Elevated c-di-AMP levels lead to decreased survival under oxidative stress and reduced staphyloxanthin production. (**A**) Survival rate of stationary growth phase bacteria after 1 h of exposure to 30 mM H_2_O_2_ in phosphate-buffered saline (PBS) was calculated relative to the control (no H_2_O_2_). (**B**) Staphyloxanthin was quantified in cultures grown for 24 h in TSB. Data display mean ± SD of at least four biological replicates, and statistical significance was assessed by unpaired Student’s *t*-test comparing WT to each strain. *P < 0.05 and **P < 0.01. PBS: phosphate-buffered saline.

A key virulence feature, promoting the survival of *S. aureus* in the host upon phagocytosis, is its resistance to oxidative stress (25). To assess how the c-di-AMP level might influence this phenotype, we quantified the survival under oxidative stress *in vitro*. Increased c-di-AMP levels led to a significantly lower survival after exposure to H_2_O_2_, whereas no statistical significance in survival was found in the *dacA_G206S_* mutant with decreased c-di-AMP levels (Fig. 2A). Resistance to oxidative stress is linked to the virulence factor staphyloxanthin, an important antioxidant in *S. aureus* (26). Staphyloxanthin levels were therefore expected to be linked with survival after oxidative stress. Indeed, staphyloxanthin levels were significantly lower in strains with elevated c-di-AMP levels (Fig. 2B).

### C-di-AMP modulates tolerance to ceftaroline and resistance to fosfomycin

**Fig 3:**
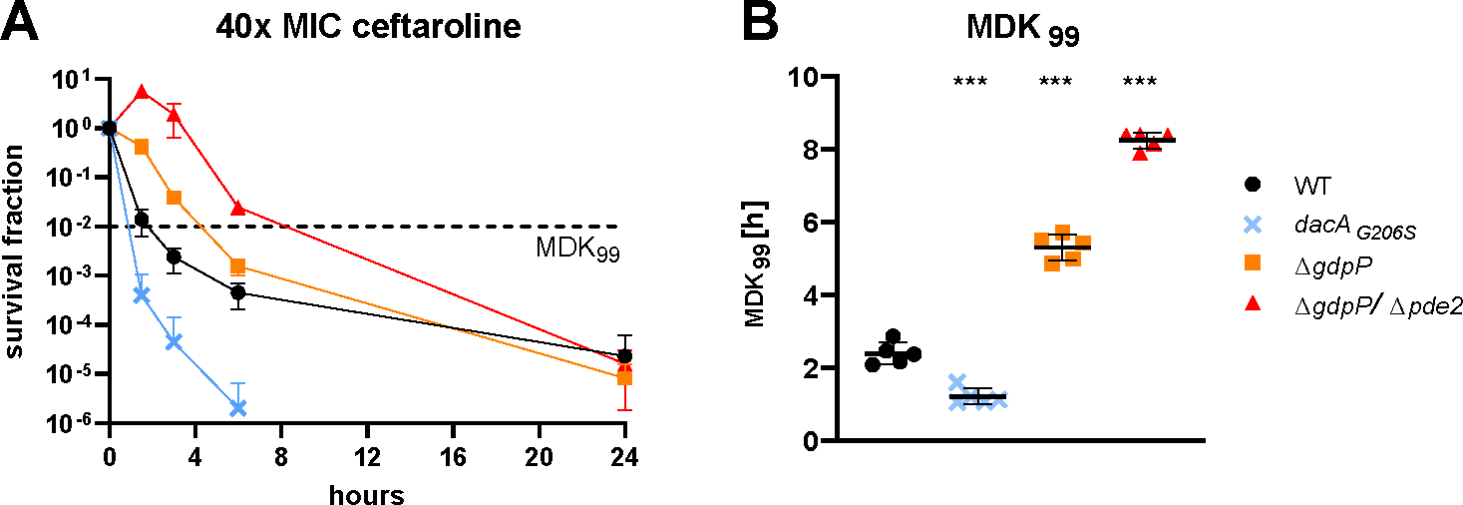
C-di-AMP modulates tolerance to ceftaroline. (**A**) The survival fraction of bacteria grown in TSB supplemented with 40× MIC ceftaroline was calculated relative to the inoculum. The horizontal dashed black line indicates a reduction of 99% (MDK_99_) of the surviving population compared to timepoint 0. (**B**) MDK_99_ values were interpolated from the killing curves in panel A. Data display five biological replicates and are presented as mean ± SD. The statistical significance of MDK_99_ values was assessed by one-way analysis of variance (ANOVA) correcting with Dunnett’s test for multiple comparisons. *P < 0.05, **P < 0.01, and ***P < 0.001.

The level of c-di-AMP positively correlates with resistance to beta-lactam antibiotics in many bacterial species (5, 11). Accordingly, the ∆*gdpP* and ∆*gdpP/*∆*pde2* mutant strains displayed a 1,000-fold increase in minimal inhibitory concentration (MIC) of the beta-lactam oxacillin (256 µg/mL) as compared to the WT strain (0.25 µg/mL), while the *dacA_G206S_* mutant was four-times more susceptible (0.064 µg/mL; Table 1, upper part). Next, we evaluated susceptibility to ceftaroline, a fifth-generation cephalosporin displaying increased affinity to the mutated penicillin-binding protein 2a (PBP2a) of MRSA strains. We observed no changes in MIC in any of the mutants (Table 1, upper part). However, a time-kill curve with 40× MIC ceftaroline revealed differences in tolerance to ceftaroline between the strains (Fig. 3A). After 1.5 and 3 h of growth in the presence of ceftaroline, the ∆*gdpP* and ∆*gdpP/*∆*pde2* mutants survived better than the WT. This difference became less pronounced after 6 h and was undetectable after 24 h. The *dacA_G206S_* strain showed a consistently lower survival rate than the WT and was completely eradicated after 6 h of 40× MIC ceftaroline treatment. In summary, tolerance quantified by minimal duration to kill 99% of the population (MDK_99_) significantly increased in the two mutants with high c-di-AMP levels and decreased in the *dacA_G206S_* mutant (Fig. 3B).

**TABLE 1:** MICs in µg/mL of various antibiotics blocking different steps in the cell wall synthesis

	Mode of action	WT	*dacA*_*G206S*_	Δ*gdpP*	Δ*gdpP*/Δ*pde2*
	Beta-lactams				
Oxacillin	Inhibits PBPs and prevents crosslinking	0.25	0.064	256	256
Ceftaroline	Inhibits PBPs with a high affinity to PBP2a and prevents crosslinking	2	2	2	1
	Cell wall active antibiotics				
Fosfomycin	Inhibits MurA	32	8	128	32
D-cycloserine	Inhibits D-Alanase ligase and racemase	64	64	64	32
Bacitracin	Prevents lipid carrier recycling	128	128	128	128
Ramoplanin	Inhibits conversion from lipid carrier I to lipid carrier II	1	1	1	1
Moenomycin	Inhibits transglycosylation	0.0625	0.0625	0.0625	0.0625

Next, we tested whether the susceptibility to cell wall active antibiotics other than beta-lactams was also influenced by c-di-AMP levels. We assessed the MICs of fosfomycin, D-cycloserine, ramoplanin, bacitracin, and moenomycin, which all interfere with different steps in the cell wall synthesis (Table 1, lower part). Altered c-di-AMP levels affected susceptibility to fosfomycin, which inhibits one of the first steps of cell wall synthesis. Susceptibility of antibiotics inhibiting later steps in cell wall synthesis was not affected (Table 1, lower part). Fosfomycin MICs increased in the ∆*gdpP* mutant and decreased in the *dacA_G206S_* strain (Table 1, lower part), while the ∆*gdpP/*∆*pde2* mutant displayed unchanged susceptibility to fosfomycin and a slightly increased susceptibility to D-cycloserine as compared to the other strains.

### Mutants with high c-di-AMP levels display a thickened cell wall

**Fig 4:**
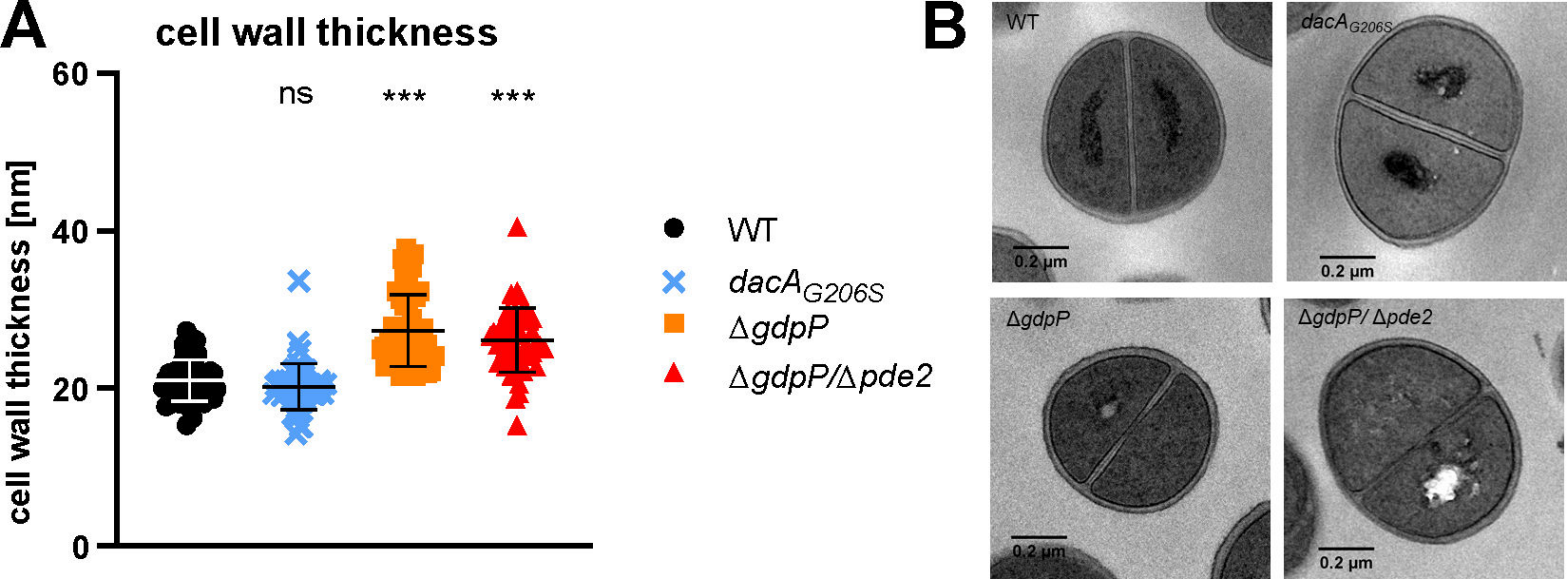
Mutants with high c-di-AMP levels display a thickened cell wall. (**A**) Cell wall thickness was determined with TEM of bacteria grown for 18 h in liquid TSB. 50–70 cells per strain were analyzed with ImageJ. Representative images of each strain are shown in (**B**). Data display mean ± SD. Each dot represents a single cell (median of five measurements per cell). Statistical significance was assessed by unpaired *t*-test comparing WT to each strain. *P < 0.05, **P < 0.01, and ***P < 0.001.

The involvement of c-di-AMP in resistance to beta-lactams and fosfomycin as well as the increased peptidoglycan crosslinking found in an *S. aureus* Δ*gdpP* strain (5) raises the question whether cell wall thickness is affected by c-di-AMP levels. Hence, we measured cell wall thickness using transmission electron microscopy (TEM). The Δ*gdpP* and Δ*gdpP*/Δ*pde2* mutants displayed a significantly thicker cell wall than the WT (27.3 ± 4.6 nm, 26.1 ± 4.1 nm, and 21.0 ± 2.6 nm, respectively; Fig. 4A and B). Lower amounts of c-di-AMP in the *dacA_G206S_* mutant did not affect cell wall thickness (20.2 ± 3.0 nm).

### High c-di-AMP levels increase the activation of the CWSS

**Fig 5:**
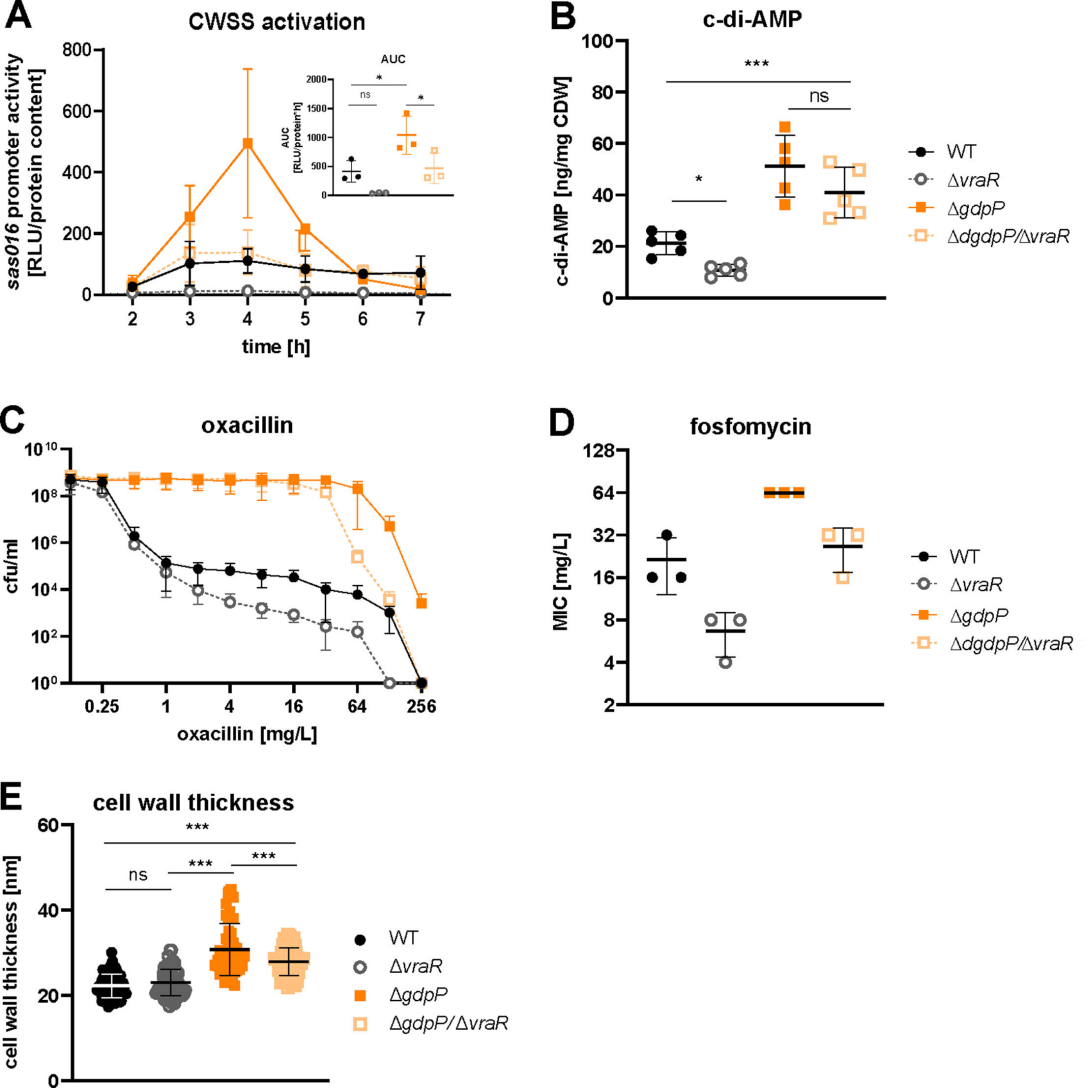
High c-di-AMP levels increase the activation of the CWSS. (**A**) CWSS activation over 7 h of growth in TSB containing tetracycline was measured via the VraR-responsive promotor of the gene *sas016* fused to a luciferase gene (17) and normalized to the protein content. The area under the curve (AUC) was calculated and is shown in the inlet. (**B**) C-di-AMP was quantified in late exponential growth cultures (OD 2) with LC-MS and normalized to the cellular dry weight (CDW). (**C**) Resistance to oxacillin is shown in a population analysis profile. (**D**) MIC of fosfomycin in TSB was measured by broth microdilution. (**E**) Cell wall thickness was determined with TEM of bacteria grown for 18 h in liquid TSB. 50–70 cells per strain were analyzed with ImageJ. Each dot represents a single cell (median of five measurements per cell). Data display mean ± SD of three (**A, C, and D**) or five (**B**) biological replicates. (**A, B, and E**) Statistical significance was assessed by one-way ANOVA correcting with Dunnett’s test for multiple comparisons. *P < 0.05, **P < 0.01, and ***P < 0.001.

Thickening of the cell wall can be caused by a misbalance between autolysis and cell wall synthesis. In *S. aureus*, cell wall synthesis increases in response to antibiotic-induced cell wall stress through differential expression of a set of over 40 genes (19). The activation of this CWSS is controlled by the three-component system VraTSR (27, 28). A link between c-di-AMP levels and CWSS expression has been shown before in an *S. aureus dacA_G206S_* strain (17). We hypothesized that increased c-di-AMP levels can lead to cell wall thickness increase and beta-lactam resistance through the activation of the CWSS and tested this hypothesis using the Δ*gdpP* mutant as an example for a strain with increased c-di-AMP levels. CWSS basal expression was assessed in the absence of antibiotic-induced stress by quantifying the activity of the *sas016* gene promoter, widely used as a proxy for CWSS activation (17, 29, 30). Confirming our hypothesis, the CWSS activity was significantly increased in the ∆*gdpP* mutant compared to the WT (Fig. 5A). To investigate whether c-di-AMP regulates CWSS expression through VraTSR signaling, we created a *∆vraR* single and a ∆*gdpP/*∆*vraR* double mutant. Deletion of *vraR* reduced CWSS activation to a minimum (non-significant difference to WT, Fig. 5A). In the ∆*gdpP/*∆*vraR* double mutant, CWSS activation was significantly reduced as compared to the ∆*gdpP* single mutant. In contrast to the *∆vraR* single mutant, the CWSS activity was comparable to the WT, indicating that c-di-AMP can activate CWSS in a VraR-independent manner (Fig. 5A). We then investigated whether VraR-dependent CWSS activation influences c-di-AMP levels. Deletion of *vraR* significantly lowered c-di-AMP concentration when compared to the WT strain, and a similar albeit not significant trend was found for the ∆*gdpP/*∆*vraR* mutant, likely due to increased variance of the ∆*gdpP* background, when compared to the ∆*gdpP* mutant (Fig. 5B). C-di-AMP levels in the ∆*gdpP/*∆*vraR* mutant remained significantly above WT level.

Increased CWSS activation confers resistance to cell wall active antibiotics (27). The elevated basal CWSS activation in the ∆*gdpP* mutant caused by high c-di-AMP levels might contribute to the resistance to oxacillin and fosfomycin. Deletion of *vraR*, and hence a reduced CWSS activation, should therefore lead to a decrease in resistance. Confirming this hypothesis, we found a decrease in resistance in the ∆*gdpP/*∆*vraR* mutant as compared to the ∆*gdpP* mutant (Fig. 5C and D). However, the double mutant remained more resistant than the WT despite a comparable basal CWSS activation level. The resistance to both antibiotics decreased in the ∆*vraR* single mutant as compared to the WT (Fig. 5C and D), in line with earlier studies (30, 31).

Finally, we investigated whether the higher basal CWSS expression level accounted for the thicker cell wall found in the ∆*gdpP* mutant. The cell wall thickness decreased significantly in the ∆*gdpP*/∆*vraR* double mutant as compared to the ∆*gdpP* mutant, 27.9 ± 3.3 nm and 30.8 ± 6.0 nm, respectively (Fig. 5E). However, the double mutant still displayed a thicker cell wall than the WT (22.3 ± 2.8 nm) and the ∆*vraR* strain (23.1 ± 3.1 nm).

In conclusion, we observed increased CWSS activation in the ∆*gdpP* strain, which partially but not completely accounted for the thickening of the cell wall and the increase in oxacillin as well as fosfomycin MIC.

## DISCUSSION

In this study, we show that c-di-AMP levels affect *S. aureus* growth, the response to oxidative stress as well as antibiotic tolerance and resistance. Antibiotic resistance and tolerance are pressing healthcare-related problems rendering effective treatment of bacterial infections increasingly difficult. Mutations in the c-di-AMP degrading phosphodiesterase GdpP have recently been found to cause resistance to beta-lactam antibiotics in clinical *S. aureus* isolates (14).

In *S. aureus*, *Streptococcus suis*, and *Streptococcus pneumoniae*, mutations in either one or both phosphodiesterases degrading c-di-AMP led to a decreased growth rate (6, 21, 32, 33). Consistent with these findings, we measured clearly reduced growth based on OD measurements and decreased colony size on agar plates in the two mutants characterized by high c-di-AMP levels. However, we could not confirm the growth defect by CFU counts, suggesting variations in cell size as a confounding factor. Our data showed that *S. aureus* cell size negatively correlated with c-di-AMP levels, leading to a substantial reduction in cell volume in the ∆*gdpP* and ∆*gdpP*/∆*pde2* mutants, characterized by high c-di-AMP levels, and to an increase in cell volume in the *dacA_G206S_* mutant, characterized by low c-di-AMP levels. This confirms earlier studies showing a decrease in cell size in the Lac* ∆*gdpP* mutant and an increase in the Lac* *dacA_G206S_* mutant (5, 22). Single-cell time-lapse microscopy revealed that the increased c-di-AMP level in the ∆*gdpP* mutant did not impact its generation time indicating that the smaller cell volume is responsible for the reduced growth detected by OD measurements. These findings are in line with a recent study showing an increase in cell size in *S. aureus* JE2 correlated with an increase in OD (34). The ∆*gdpP*/∆*pde2* and *dacA_G206S_* mutants showed a longer generation time indicating a c-di-AMP optimum concentration range for unimpaired growth. Similar results have been found in *Streptococcus pyogenes*, where a ∆*gdpP*/∆*pde2* and a *dacA_G206S_* mutant, but not the ∆*gdpP* single mutant, showed an increased doubling time in the liquid medium during exponential growth (35).

Although mutations leading to increased c-di-AMP levels have been found to be relevant for beta-lactam resistance in clinical isolates, it is still unclear how c-di-AMP affects the virulence of these isolates. The antioxidant staphyloxanthin is an important virulence factor that protects *S. aureus* from oxidative stress, encountered during infections in the host environment. We observed lower levels of staphyloxanthin in the two strains with high c-di-AMP levels. Matching the decreased staphyloxanthin concentrations, survival after exposure to H_2_O_2_ was reduced in strains with increased c-di-AMP levels. This confirms previous findings showing a downregulation of the superoxide dismutase *sodM* in an *S. aureus ΔgdpP* mutant, explaining the decreased ability to cope with oxidative stress (21).

The exact mechanism how c-di-AMP mediates resistance to beta-lactams is not yet known. In general, resistance to beta-lactams in *S. aureus* is mediated by PBP2a encoded by the *mecA* gene (1) or by PBP4 in *mecA*-negative strains (12, 13). Increased *pbp4* expression was found in an *S. aureus ΔgdpP* strain with increased beta-lactam resistance (21). In contrast, a recent study reported unchanged *pbp4* expression in beta-lactam-resistant clinical *S. aureus* isolates lacking *mec*A (14). However, the majority of these *mecA*-negative clinical isolates had acquired mutations in *gdpP*, indicating a clinical relevance of strains with increased c-di-AMP levels. Confirming earlier studies, we found drastically increased MICs for the beta-lactam oxacillin in the *ΔgdpP* and *ΔgdpP/*Δpde2 mutants and a decreased MIC in the *dacA_G206S_* mutant (5, 14, 15, 34, 36). In clinics, cephalosporins are often used to treat MRSA infections as they exhibit high affinity to the MRSA-specific PBP2a. MICs to ceftaroline, a fifth-generation cephalosporin preventing peptidoglycan crosslinking, remained unchanged in the *ΔgdpP* and *ΔgdpP/*Δpde2 mutants, suggesting that increased peptidoglycan crosslinking does not affect resistance to this antibiotic (5). This is supported by Ba et al. showing increased resistance to oxacillin but not to cefoxitin, a second-generation cephalosporin, in clinical *mecA*-negative MRSA isolates harboring a *gdpP* mutation (15). Similarly, unchanged susceptibility to ceftaroline was found in an *S. aureus* strain with a mutated *clp*, a protease involved in various cell wall processes such as peptidoglycan crosslinking, beta-lactam resistance, and cell size, leading to a Δ*gdpP*-like phenotype (37). However, despite having similar MICs, time-kill curves showed prolonged survival and an increased MDK_99_ for the Δ*gdpP* and Δ*gdpP/*Δ*pde2* mutants. As antibiotic tolerance is defined by an increase in MDK_99_ (38), high c-di-AMP levels seem to promote tolerance to ceftaroline. This is in line with a study showing that certain mutations in the staphylococcal *gdpP* gene lead to beta-lactam and glycopeptide antibiotics tolerance (36). Not all *gdpP* mutations found in clinical isolates conferred beta-lactam resistance; therefore, the question remains whether those mutations might cause tolerance and thereby pave the way to resistance development (14, 16). A role of c-di-AMP in multi-drug tolerance evolution has recently been proposed in *Mycobacterium smegmatis* (39). Our data suggest that increased c-di-AMP levels delay the ceftaroline effect by promoting tolerance, underlining the difficulties in eradicating these bacteria with certain antibiotics.

While the influence of c-di-AMP on the resistance to beta-lactams has been described, data about resistance to other cell wall active antibiotics are still scarce. We tested five antibiotics (fosfomycin, D-cycloserine, bacitracin, ramoplanin, and moenomycin) of different classes inhibiting distinct cell wall synthesis steps. Only resistance to fosfomycin differed between the strains, increasing in the *ΔgdpP* mutant and decreasing in the *dacA_G206S_* mutant. Since fosfomycin inhibits MurA, an enzyme catalyzing the formation of the peptidoglycan precursor UDP-MurNAc (40, 41), these observations suggest that c-di-AMP affects the first steps of cell wall synthesis. However, the fosfomycin MIC remained unchanged in the Δ*gdpP*/Δ*pde2* double mutant compared to the WT. A study in *S. pyogenes* revealed distinct Δ*gdpP* and Δ*pde2* phenotypes regarding growth and virulence, suggesting different cellular roles of the two phosphodiesterases (35). In contrast to GdpP, Pde2 mainly degrades pApA, the product of c-di-AMP degradation by GdpP. In a previous study, an accumulation of pApA was found in an *S. aureus* Δ*gdpP*/Δ*pde2* mutant, and it has been suggested that pApA acts as a nanoRNA source and thereby influences the priming of transcription and ultimately gene expression (6, 35, 42).

Resistance to cell wall active antibiotics often correlates with a thicker cell wall in *S. aureus* (12, 37, 43), and c-di-AMP was shown to be involved in cell wall homeostasis (35, 44). We hypothesized that the increased antibiotic resistance and tolerance found in the mutants characterized by high c-di-AMP levels correlate with a thicker cell wall. Confirming our hypothesis, we detected an increase in cell wall thickness in the *ΔgdpP* and *ΔgdpP/*Δ*pde2* mutants. Whether the thicker cell wall is directly responsible for the increased antibiotic resistance and tolerance still needs to be elucidated.

Cell wall thickness is influenced by autolysis, peptidoglycan crosslinking, and cell wall synthesis (45, 46). In *S. aureus*, cell wall synthesis can increase upon cell wall stress induced by antibiotics. Such stress triggers the three-component regulatory system VraTSR that regulates the expression of a cluster of more than 40 genes involved in cell wall synthesis, known as CWSS (18, 47). The exact signal triggering the VraTSR cascade is still unclear (48). It was shown that reduced c-di-AMP levels correlate with a decreased basal CWSS activation in two different *S. aureus* strains carrying the *dacA_G206S_* mutation (17). We therefore hypothesized that high c-di-AMP levels could trigger CWSS activation in the absence of exogenous stressors and lead to an increase in cell wall thickness, essentially locking the bacteria in a stress-related phenotype and decreasing susceptibility to cell-wall active antibiotics. In line with our hypothesis, we detected increased CWSS activation in the Δ*gdpP* mutant. In the Δ*vraR* single mutant, CWSS activation was abolished, confirming an earlier study (27); however, the deletion of *vraR* in the Δ*gdpP* mutant reduced CWSS activation only to WT level. This observation suggests that c-di-AMP activates CWSS in a VraR-independent manner. However, further investigations are needed to confirm a VraR-independent CWSS activation and find the responsible mechanisms. The increased CWSS activation triggered by c-di-AMP might contribute to a thicker cell wall and increased resistance to oxacillin and fosfomycin as the resistance and cell wall thickness phenotype of the Δ*gdpP/*Δ*vraR* double mutant lies between the WT and the Δ*gdpP* single mutant. Additionally, the loss of *vraR* lowered c-di-AMP levels in the WT strain and the Δ*gdpP* mutant, indicating a role of *vraR* in c-di-AMP homeostasis. An opposite effect was found in *Enterococcus faecalis*, where a *liaR*, which is a *vraR* homolog, mutant was characterized by increased c-di-AMP levels without changes in the expression of the enterococcal *dacA* and *gdp*P homologs (49), suggesting a species-specific interaction between c-di-AMP levels and the VraTSR regulatory system.

In conclusion, our study shows that a previously described growth defect in *S. aureus* producing high c-di-AMP levels is mainly attributable to smaller cell size. High levels of c-di-AMP impaired virulence by reducing resistance to oxidative stress and staphyloxanthin production, resulted in a thicker cell wall promoting increased resistance to oxacillin and fosfomycin as well as tolerance to ceftaroline and lead to higher CWSS activation. Taken together, we report the importance of studying the role of c-di-AMP in promoting antibiotic tolerance and increasing cell wall thickness, potential mechanisms leading to the development of antibiotic resistance, with the aim of streamlining antibiotic treatment and ultimately improve patient care.

## MATERIALS AND METHODS

### Bacterial strains and growth conditions

Bacterial strains and plasmids used in this study are listed in Table S2. Strains were grown in TSB (BD) at 37°C shaking or on TSB agar plates. Tetracycline was used at 10 µg/mL, chloramphenicol at 10 µg/mL, and anhydrotetracycline at 0.2 µg/mL when appropriate. Overnight cultures were grown for 14 to 18 h, diluted 1:50 in fresh medium, and grown for 1.5 h to obtain logarithmically growing bacteria.

### Plasmid transduction and genetic manipulations

The VraR deletion mutant was constructed by inserting two stop codons by homologous recombination at the second and third codon of the *vraR* gene using a pKOR1 plasmid (30). pKOR1-VraR::stop was transduced into the LAC* WT and the Δ*gdpP* mutant background using phage 80α and chromosomally integrated as previously described (50). LAC* *dacA_G206S_* was constructed using the pKOR1-*dacA-*SNP plasmid from references (17). The plasmid was transduced into LAC* WT using phage 80α and chromosomally integrated. The manipulated regions were checked by Sanger sequencing. The *sas016* promoter luciferase fusion plasmid p*sas016*_p_-*luc* (*29*) was transduced into LAC* WT, Δ*gdpP*, and Δ*gdpP/*Δ*vraR* mutant background using phage 80α.

### Colony growth dynamics quantification

Bacteria from stationary or logarithmic growth phase with or without exposure to H_2_O_2_ stress (30 mM H_2_O_2_, 1 h) were diluted to reach 50–150 CFUs per plate (Columbia agar plates with 5% sheep blood, COS, Biomérieux). Plates were incubated at 37°C, and images were acquired with a Canon EOS 1200D reflex cameras every 10 min for 48 h. Cameras were triggered by Arduino Uno board and optocouplers. Colony growth dynamics were analyzed as previously described using ColTapp (51).

### Determination of cell volume and generation time using single-cell microfluidics

The size and generation time (time from birth to division of a given cell) of single bacterial cells were determined using a mother-machine microfluidics device combined with timelapse microscopy. Device design and fabrication have been described elsewhere (52). In brief, cells were embedded in chambers of height 0.93 µm, length 25 µm, and width ranging from 1.2 to 1.6 µm perpendicular to a flow channel. Bacteria were grown overnight in TSB supplemented with 0.01% Tween20 to avoid clumping. The cultures were diluted 1:100, regrown for 2 h, and then washed in TSB 0.1% Tween20 and concentrated in TSB 0.01% Tween20. Bacteria were then loaded into the microfluidics chip with a pipette. The outlet and inlets were connected with tubings (Adtech, PTFE, inner diameter: 0.3 mM, outer diameter: 0.76 mM) to a waste collection container and to a syringe pump (KF Technology, NE-1600) providing fresh TSB 0.01% Tween20 at a flow rate of 0.5 mL/h. The chip was placed inside the heated chamber (37°C) of the fully automated inverted microscope (Olympus IX83 P2ZF) controlled via CellSense 3.2. After letting the bacteria recover for 4–5 h from the loading procedure, imaging was started. Phase contrast (200 ms exposure, 130 intensity IX3 LED) images were acquired every 2 min for 5 h with a 100×, numerical aperture 1.3 oil phase objective (Olympus) and an ORCA-flash 4.0 sCMOS camera (Hamamatsu). A custom MATLAB (MathWorks) script was used to register image sequences, detect filled chambers, and crop out single-chamber images. On these, single-cell segmentation was performed with a retrained deep-learning StarDist 2D model (53). The segmentations were then injected in the retrained deep-learning cell-tracking model of DeLTA 2.0 (54). DeLTA 2.0 output was filtered to include only cells for which we observed a mother cell and a division event. We reported the time between division events as generation time and mean cell volume over the lifetime of a cell. Cell volume was estimated as described before (55) using equation 1 (with a and b corresponding to the longer and shorter semi-axes, respectively).

(1) Volume = $\frac{4}{3}$ πab^3^

### Susceptibility to H_2_O_2_

Stationary growth phase bacteria were diluted to approximately 5 × 10^6^ CFU/mL in phospate-buffered saline (PBS) and incubated for 1 h at 37°C with 30 mM H_2_O_2_. Bacterial survival relative to the control (no H_2_O_2_) was determined by plating serial dilutions on blood plates (Columbia agar plates with 5% sheep blood, COS, Biomérieux) in order to inactivate any residual H_2_O_2_.

### Staphyloxanthin production

Staphyloxanthin production was quantified as described before (56) with a few modifications. In short, 2 mL of a stationary growth phase culture (24 h, TSB) were harvested by centrifugation (maximum speed, 5 min, and room temperature) and washed with PBS. The washed pellet was completely dried with a centrifuge concentrator (Eppendorf) for 1.5 h at 30°C. After vortexing the pellet in 20 µL PBS containing glass beads, 180 µL of methanol was added, and the samples were kept shaking (1,000 rpm) at 55°C for 4 min. After centrifugation (maximum speed, 10 min), the absorbance of the supernatant was measured with a plate reader (Tecan Infinite M Nano) at OD_465nm_.

### Transmission electron microscopy

Overnight bacterial cultures grown in TSB were washed in PBS and resuspended in cacodylate buffer (0.1 M) with 2.5% glutaraldehyde. The samples were processed for image analysis as previously described (57). Images were taken with a 120-kV transmission electron microscope (FEI Tecnai G2 Spirit) equipped with two digital CCD cameras. Cell wall thickness was assessed with ImageJ in 50–70 cells per strain by manually measuring the observed cell wall in zones that are not in proximity of the division septum. The median of five measurements per cell was used to calculate the mean cell wall thickness of each strain.

### Minimal inhibitory concentrations

MICs were determined in TSB with the broth microdilution method according to EUCAST guidelines (58). Oxacillin MICs were assessed using commercial MIC test strips (Etest, Biomérieux). MICs were assessed in biological triplicates.

### Oxacillin population analysis profiles

Antibiotic resistance population profiles were determined by plating appropriate dilutions of overnight culture, ranging from undiluted to 10^−7^, on TSB agar plates containing increasing concentrations of oxacillin. Plates were incubated at 37°C and CFU/mL were determined after 48 h.

### Time-kill curves

Bacteria were grown to the logarithmic growth phase as described above, washed, and inoculated in TSB containing ceftaroline at a concentration of 40× MIC (inoculum 5 × 10^5^ CFU/mL). After 1.5, 3, 6, and 24 h, bacterial numbers were assessed by plating, and the survival fraction was calculated relative to the inoculum. The values for the minimal duration time to kill 99% of the population (MDK_99_) were interpolated using linear regression and tested for statistical significance by ANOVA correcting with Dunnett’s test for multiple comparisons.

### Luciferase reporter assay

To measure the luciferase activity of the *sas016* promoter (29) over different growth stages, cultures were inoculated at OD_600nm_ 0.05, samples were collected after 2, 3, 4, 5, 6, and 7 h of growth and centrifuged, and pellets were stored at −20°C until further use. Pellets were defrosted and resuspended to OD_600nm_ 10 in PBS. Immediately after the addition of the luciferase substrate (Promega) at a 1:1 ratio, luminescence was measured with a plate reader (Spectramax i3, Molecular Devices) using the standard settings for luminescence measurements. Luminescence values were normalized to the protein content at OD10. Protein content was assessed with the Pierce BCA protein assay kit (Thermo Scientific) according to the manufacturer’s instructions. The area under the curve (AUC) was calculated for each replicate and tested for statistical significance by ANOVA followed by pairwise comparisons with correction for multiple comparisons.

### C-di-AMP quantification

C-di-AMP was quantified as described previously with some modifications (17). Briefly, bacteria were grown to log phase (Table S1) or to approximately OD_600nm_ 2 (Fig. 5), collected by fast filtration, and immediately quenched (−20°C cold acetonitrile 60%, formic acid 0.5 M 20%, and methanol 20%). C-di-GMP (Biolog) was added as an internal standard. Samples were sonicated four times for 20 s before being snap frozen in liquid nitrogen and lyophilized. Analysis was performed on a Thermo Ultimate 3,000 UHPLC system (Thermo Scientific, CA, USA) coupled to Thermo OExactice plus instrument (Thermo Fisher Scientific, Waltham, MA, USA) equipped with a heated electrospray ionization probe. LC separation was carried out at a flow rate of 500 µL/min applying a quaternary gradient, summarized as a binary gradient. Solvent A was 10 mM ammonium formate adjusted to pH 8.1, and solvent B was 10 mM ammonium formate adjusted to pH 8.1 in methanol to water in a ratio of 80:20. Gradient B was as follows: 0 min, 2.5%; 4.2 min, 28.8%; 8.9 min, 97.5%; 9.9 min, 97.5%; 10.0 min, 2.5%; 13.1 min, 2.5%. MS spectra were acquired in the positive Fourier transform mass spectrometry (FTMS) mode at a mass resolution of 70,000 (m/z = 200). Five biological replicates per strain were analyzed. For normalization (Table S1), total protein content was assessed with the Pierce BCA protein assay kit (Thermo Scientific) according to the manufacturer’s instructions. C-di-AMP concentration was normalized to the internal standard (A_internal_) and OD/total protein ratio (OD_perTP_) using equation (2).

(2) A_norm_ = $\frac{Asample}{Ainternal}*\frac{ODperTP}{OD600*Vsample}$ .

C-di-AMP concentration of the *vraR* mutant strains (Fig. 5) was normalized to the cellular dry weight as described before (17).

### Statistical analysis

Data were analyzed with GraphPad Prism 8.0, R 4.3, and RStudio 1.3. Cell volume, generation time, and colony size were assessed with a mixed effects model using a random intercept term for the biological replicate followed by estimated marginal means post-hoc tests (multivariate *t*-distribution based *P*-value correction) (59). All other experiments were analyzed with either unpaired Student’s *t*-tests or ANOVA followed by Dunnett’s test for multiple comparisons.

## SUPPLEMENTAL MATERIAL

Fig. S1, Tables S1 and S2, legend to Movie S1, and supplemental methods spectrum.02788-23-s0001.pdf Supplemental Material.

Movie S1C_ΔgdpP spectrum.02788-23-s0002.avi Representative movies of cells growing in a mother-machine microfluidic device.

Movie S1D_∆gdpP/∆pde2 spectrum.02788-23-s0003.avi Representative movies of cells growing in a mother-machine microfluidic device.

Movie S1B_dacAG206S spectrum.02788-23-s0004.avi Representative movies of cells growing in a mother-machine microfluidic device.

Movie S1A_WT spectrum.02788-23-s0005.avi Representative movies of cells growing in a mother-machine microfluidic device.

ASM does not own the copyrights to Supplemental Material that may be linked to, or accessed through, an article. The authors have granted ASM a non-exclusive, world-wide license to publish the Supplemental Material files. Please contact the corresponding author directly for reuse.
